# Tailored Dispersion of Spectro‐Temporal Dynamics in Hot‐Carrier Plasmonics

**DOI:** 10.1002/advs.202205434

**Published:** 2023-01-19

**Authors:** Andrew S. Kim, Mohammad Taghinejad, Anjan Goswami, Lakshmi Raju, Kyu‐Tae Lee, Wenshan Cai

**Affiliations:** ^1^ School of Electrical and Computer Engineering Georgia Institute of Technology Atlanta GA 30332 USA; ^2^ School of Materials Science and Engineering Georgia Institute of Technology Atlanta GA 30332 USA

**Keywords:** hot carriers, nanophotonics, nonlinear optics, optical modulation, plasmonics, ultrafast optics

## Abstract

Ultrafast optical switching in plasmonic platforms relies on the third‐order Kerr nonlinearity, which is tightly linked to the dynamics of hot carriers in nanostructured metals. Although extensively utilized, a fundamental understanding on the dependence of the switching dynamics upon optical resonances has often been overlooked. Here, all‐optical control of resonance bands in a hybrid photonic‐plasmonic crystal is employed as an empowering technique for probing the resonance‐dependent switching dynamics upon hot carrier formation. Differential optical transmission measurements reveal an enhanced switching performance near the anti‐crossing point arising from strong coupling between local and nonlocal resonance modes. Furthermore, entangled with hot‐carrier dynamics, the nonlinear correspondence between optical resonances and refractive index change results in tailorable dispersion of recovery speeds which can notably deviate from the characteristic lifetime of hot carriers. The comprehensive understanding provides new protocols for optically characterizing hot‐carrier dynamics and optimizing resonance‐based all‐optical switches for operations across the visible spectrum.

## Graphical abstract

Dynamic optical properties in plasmonic nanostructures upon excitation of hot‐carriers are utilized for building ultrafast all‐optical switches, an optical counterpart of transistors. Angle‐resolved pump‐probe spectroscopy performed on nanostructures supporting optical resonances with angle‐dependent spectral locations reveals novel strategies to increase the switching efficiency and expedite the transient process. The new design rules offer pathways for performance enhancement of all‐optical switches.

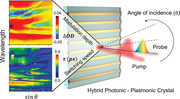

## 1 Introduction

The everlasting need for fast and power efficient computation schemes makes optical data processing a promising candidate for replacing conventional methods using electronics. Instead of electrons, optical computing utilizes photons as the carrier of information to potentially enable a faster operation speed with less energy consumption. To achieve a complete set of device components for the implementation of optically tunable platforms, an optical counterpart of transistors is essential to build logic circuits. In particular, achieving all‐optical switching–controlling the attributes of light with light—stands out as a primary goal toward the realization of a data processing unit that receives, computes, and transmits optical signals. In a switch configuration, transistors act as nonlinear and active elements transitioning between two logic states which occur upon the arrival of an electronic command signal. In principle, a similar strategy can be implemented in optics by employing the nonlinear interaction of light with optical media incorporated in the compartment of optical resonators. The optical Kerr nonlinearity is perceived as a key nonlinear effect that enables the tuning of refractive indices in optical materials to facilitate all‐optical control over the interaction of an information‐carrying input light with an optical resonator.^[^ ^1^, ^2^ ^]^ Despite the diverse portfolio of optical materials with large Kerr nonlinearities, noble metals have enticing advantages over other counterparts. Indeed, nanostructured noble metals comprise the foundation of plasmonics, where the optical electric‐field localization to a nanoscale regime strongly boosts the effective Kerr nonlinearity of noble metals, rendering them as a primary candidate for the realization of efficient all‐optical switches with a nanoscale footprint. Such a unique opportunity has been the motivation behind the surge of publications on active plasmonics to explore and understand the interplay between optical properties of plasmonic resonators and photophysical phenomena in noble metals. The current span of literature is enriched by a variety of strategies to achieve all‐optical switching of the intensity, phase, polarization, and frequency of light by employing various plasmonic designs and material architectures.^[^ ^3^, ^4^, ^5^, ^6^, ^7^, ^8^, ^9^, ^10^, ^11^ ^]^

From a microscopic perspective, the Kerr‐type optical nonlinearity of plasmonic metals is linked with the energy evolution of hot electrons.^[^ ^2^ ^]^ In the literature, the term “hot electrons” is often employed to describe electrons in a solid with an energy larger than that of the room temperature.^[^ ^12^, ^13^, ^14^, ^15^ ^]^ In metallic nanostructures, the nonradiative decay of plasmons enables interband and intraband electronic transitions that yield a dense population of hot electrons in the conduction band of metals.^[^ ^16^, ^17^, ^18^ ^]^ The optical manipulation of the effective temperature and density of electrons, mediated by the generation of hot electrons, significantly changes the dielectric permittivity of metals,^[^ ^19^ ^]^ which manifests itself in the form of a large Kerr coefficient in nanostructured noble metals. In fact, the large Kerr nonlinearity in noble metals allows non‐invasively studying the dynamics of its origin with ease, which itself is an important subject considering the vast applications hot carriers offer. Hence, investigating hot‐carrier dynamics and building efficient all‐optical switches form a bilateral relation. Contrary to fully coherent nonlinear optical interactions, hot‐carrier‐driven nonlinear effects can be tuned via external means to deliver desired optical functionalities in compliance with a set of prescribed performance measures. On the flip side, however, this enforces shortcomings for achieving fast switching speed where the involvement of hot carriers often limits the modulation speed to the lifetime of temporal dynamics of their excess energy. For instance, gold possesses a strong Kerr nonlinear response at the spectral vicinity of the interband transition threshold that scales with the effective electron temperature. However, since the elevation of electron temperature notably lowers the scattering rate between electrons and phonons, maximizing the modulation depth in a gold‐based plasmonic optical switch often comes at the cost of the modulation speed. To overcome this innate trade‐off, activating a hot‐electron transport pathway at the interface of plasmonic metals and dielectric materials has been proposed as an alternative relaxation pathway that weakens the dominant role of the electron‐phonon scattering on the modulation speed of optical switches.^[^ ^7^ ^]^ Nonetheless, a comprehensive study on the correlation between the modulation performance of a hot‐electron‐driven optical switch and the optical resonances also has the potential to open up opportunities for expediting the switching speed, which benefits the endeavor of overcoming the trade‐off limits. From an alternative perspective, if one were to utilize plasmonic nanostructures as a media to investigate the temporal characteristics of hot‐carrier dynamics, the correlation between the switching speed and optical resonances also becomes of great importance as geometrically induced optical resonances in plasmonic nanostructures can cause the recovery speed of the transient optical response to deviate from the actual timescale of hot‐carrier relaxation. Such knowledge thus empowers both the fundamental understanding of hot‐carrier dynamics and the engineering capabilities of spectro‐temporal attributes of hot‐carrier‐based active optical elements.

In this study, we explore the transient behavior of a hybrid photonic‐plasmonic crystal in response to the formation of hot electrons in a 1D array of metallic nanostripes integrated with a dielectric slab. The periodic arrangement of nano‐optical resonators offers the versatility to tailor the collective optical response, where meticulous selection of material and geometrical parameters further enables emphasis on a desired nonlinear process that comprises the Kerr nonlinearity.^[^ ^7^, ^20^, ^21^, ^22^, ^23^, ^24^, ^25^ ^]^ This allows optical properties of interest to be tuned with enhanced switching speeds and efficiencies. The designed platform supports two sets of photonic and plasmonic modes that are independently accessible via orthogonal polarization states of light and are spectrally tunable via the manipulation of the in‐plane light momentum. Specifically, photonic modes were designed to exhibit a bandgap near the edge of the d‐band transition energy levels of gold. Here, the spectral overlap between the structural and material bandgaps provides a unique opportunity to employ the hot‐electron driven nonlinear response of gold, and dynamically tune the photonic bandgap (PBG) of the hybrid platform in an all‐optical and ultrafast fashion. In addition, we capitalize on the versatility of the devised hybrid system to comprehensively probe the correlation between the plasmonic/photonic resonance behaviors and the relaxation dynamics of the perturbed light transmission through the device. Our findings reveal enhanced switching performances under specified probing conditions and show that the relaxation dynamics of the perturbed optical response strongly depend on the relative spectral distance of the investigated wavelength with respect to the spectral locations of plasmonic/photonic resonance modes, which depends on the in‐plane momentum of light. Experimental observations reveal an expedited recovery process away from the resonance wavelength with the possibility of an all‐optical switching speed even faster than the characteristic timescales for the relaxation of photocarriers. The broken correspondence between the transient optical response and hot‐carrier dynamics therefore offers new routes to accelerating the switching speed, which alleviates number of constraints including geometrical design, material selection, and pump conditions previously required for achieving expedited carrier relaxation dynamics. The comprehensive outcome of this study sets new principles for optically assessing the relaxation dynamics and may serve as a benchmark for optimizing resonance‐based all‐optical switches across the visible spectrum.

## 2 Results

### 2.1 Experimental Procedure

**Figure 1:**
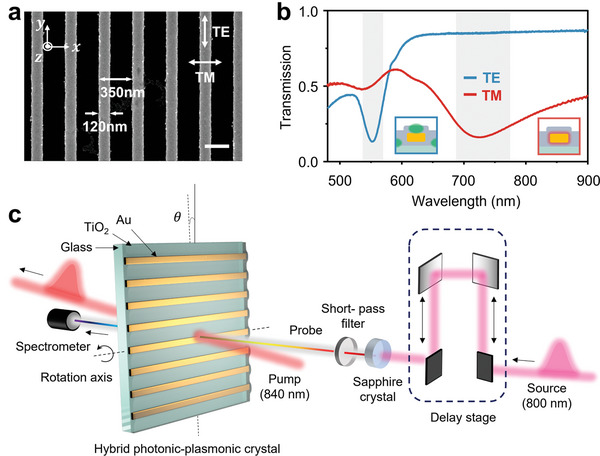
Illustration of the fabricated device and the experimental setup. a) Scanning electron microscope image of the sample. The naming conventions for the coordinates and polarization directions are also included. The scale bar equals to 250 nm. b) Linear optical transmission spectra under normal incidence for TE and TM polarizations. The insets conceptually describe the field enhancement profile for each resonance mode, where the TE resonance originates from the lattice structure while the TM resonance stems from the individual nanostripe. c) Schematic of the simplified angle‐resolved transient pump‐probe spectroscopy setup. A pulsed laser with a center wavelength of 800 nm is used to generate both pump and probe pulses. Narrowband pump pulses with a center wavelength of 840 nm are generated through an optical parametric amplifier, and supercontinuum probe pulses are generated through a nonlinear crystal. The transient response map can be obtained by continuously scanning the delay between the pump and probe pulse through a delay line. Both the linear and transient optical characterization were performed at the same setup.

The structure of interest is a 1D plasmonic nanograting made of gold (Au) embedded in a slab of titanium dioxide (TiO_2_). **Figure** **1**a shows a representative scanning electron micrograph of the fabricated structure (see the Experimental Section for details). As evident from the static optical transmission response (Figure 1b), the devised structure exhibits two polarization‐sensitive resonance modes with distinct electromagnetic natures. Under normal illumination, TE‐polarized light excites a spectrally narrow resonance mode at a center wavelength of 552 nm, while a TM‐polarized beam grants access to a broad resonance feature near 720 nm wavelength. These two modes are fundamentally different in the sense that the narrow TE‐polarized resonance is essentially a nonlocal photonic mode formed inside the TiO_2_ slab via diffractive coupling,^[^ ^26^, ^27^ ^]^ whereas the TM‐polarized mode is a localized surface plasmon resonance (LSP) with a resonance wavelength that is primarily influenced by the width of the gold nanostripes. The photonic mode at the 552 nm wavelength is a guided mode resonance (GMR) and is accessible via free space illumination owing to the integration of the TiO_2_ slab with the periodic gold array.^[^ ^28^ ^]^ The polarization selective accessibility to the resonance mode and the small thickness of the dielectric slab rule out the possibility of the resonance being a Fabry‐Perot mode. The nature of this photonic mode being a GMR mode becomes more evident once we investigate the near field distribution or introduce an in‐plane momentum to the impinging light, which is presented later in this work. The spatial confinement of the GMR mode within the TiO_2_ slab makes it notably immune from the absorption loss of gold at the spectral vicinity of the interband electronic transitions (i.e., *λ*<650 nm), enabling the formation of a relatively narrow resonance at 552 nm wavelength. This capability is important once we notice that around the GMR mode the optical Kerr nonlinearity of Au is strongest. Therefore, by optically tuning the refractive index of Au through a pump beam one can effectively control the coupling efficiency of free space light into the GMR mode.

Figure 1c depicts the setup for the angle‐resolved optical characterization of our structure, which was used to perform both static and transient optical measurements. The setup follows a standard pump‐probe configuration, where ultrashort pulses of a narrowband pump beam are introduced to generate hot electrons inside the nanograting structure. The resultant change in the optical response is then monitored by delayed pulses of a broadband probe light in the visible spectrum. By continuously scanning the delay time between the pump and probe beams, we explore the temporal evolution of the optical properties of the 1D crystal in response to the energy evolution of hot electrons. Prior to the transient measurements, the linear optical response was obtained by only using the probe light. We study the interplay between momentum of the incident light and the optical properties of the 1D hybrid photonic‐plasmonic crystal by controlling the angle of incidence (AOI) *θ* in the *x*–*z* plane, which changes the in‐plane momentum of light in the *x*–direction. The *x*‐component of the incident wavevector can be expressed as *k_x_* = *k*_0_sin(*θ*), where *k*_0_ stands for the free space wavevector. Because the beam path of the pump and probe light were both fixed, the sample was rotated about an axis parallel to the Au nanostripes instead, with the rotation axis passing through the center of the sample to maintain a fixed illumination spot on the sample.

### 2.2 Static Optical Response

**Figure 2:**
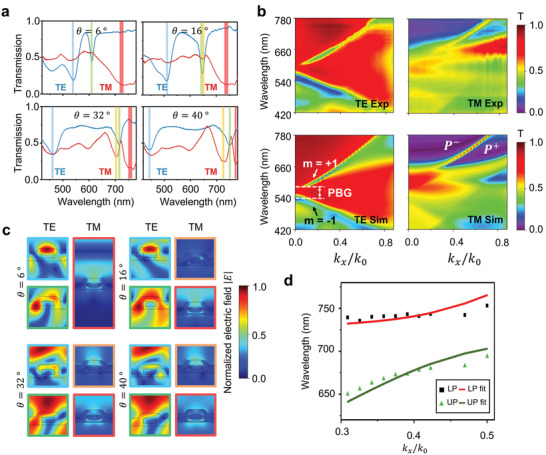
Static optical response of the nanograting. a) Measured linear optical transmission at different AOIs under both TE and TM polarization. b) Measurements and numerical simulations of the angular dispersion of the optical transmission under TE and TM polarization. For TE polarization, two resonance bands are observed with a photonic bandgap, where the resonance bands are labeled by the corresponding diffraction order of the incident light. Two polariton branches are observed at higher AOIs under TM polarization which are labeled on the plot. c) Near field profile of the nanograting for different resonance modes under varying AOIs. The color coding at the perimeter of each near field profile provides information on the corresponding resonance modes, which are visually represented through the colored bars given in Figure 2a. d) Numerical fitting of the obtained spectral locations of polariton modes to a coupled‐oscillator model.

Measured optical transmission responses of the structure for a few selected AOIs under both TE‐ and TM‐polarized illuminations are given in **Figure** **2**a. Unlike the optical response under normal incidence (Figure 1b), we observe an additional transmission dip under the TE illumination. As we increase the AOI, a gradual increase in the spectral distance between the two transmission dips can be observed. This is a characteristic behavior of the GMR mode which occurs when the following momentum matching condition is satisfied:^[^ ^26^ ^]^

(1) $$\left|k_{x}n_{\mathrm{eff}}-m\frac{2\pi }{p}\right|=\beta_{\mathrm{TE}},\;\left(m=\pm 1,\pm 2\cdots \right)$$

The left side of the equation is the summation between the effective in‐plane momentum of the impinging light within the plane of the 1D crystal and the reciprocal lattice vector 2*π*/*p* of the periodic array with multiple orders. The periodicity of the nanograting is denoted as *p* and *n*_eff_ is the effective refractive index of the waveguiding layer. The material parameter *n*_eff_ is not equal to the intrinsic refractive index of TiO_2_ due to the embedment of the Au nanograting, and differs from the TE waveguiding mode index which is accounted for in the right‐hand side of Equation (1). *β*_TE_ is the wavevector of the TE‐polarized guided mode, and the absolute value sign indicates the propagation direction independence. The in‐plane momentum dependence results in a monotonic spectral shift for the two transmission dips toward opposite directions. For TM‐polarized light, we notice that an additional transmission dip emerges near 650 nm which we attribute to the nonlocal lattice plasmon (LP) mode. The LP mode occurs when the incoming light satisfies the Rayleigh anomaly condition, and therefore a spectral shift proportional to the in‐plane momentum of light is observed.^[^ ^29^, ^30^, ^31^ ^]^ As the LP mode resonance spectrally approaches the LSP mode at higher AOIs, coupling between the bright (LSP) and dark (LP) mode occurs and thus a Fano‐type lineshape shows up^[^ ^31^, ^32^, ^33^ ^]^ where a sharp transmission window is observed.

The angular dispersion of the optical transmission is plotted in Figure 2b. Numerical simulation results using COMSOL are plotted together as well, where a fair resemblance between the spectral features is witnessed. The refractive indices of TiO_2_ and Au used in the simulation were retrieved from ellipsometry measurements and the literature, respectively.^[^ ^34^ ^]^ At first glance we see two distinct resonance bands for TE polarization, which are the GMR bands. The resonance band that red (blue) shifts upon increasing AOI corresponds to the waveguide mode coupled with positive (negative) first order diffraction which can be traced by plugging in +1 and −1 as the value for *m* in Equation (1). As the in‐plane momentum of the incident light grows the positive first order band emerges, leading to the formation of a PBG. Under TM‐polarized illumination, the dispersion map of the light transmission through the hybrid structure exhibits two distinct resonance bands between 700–800 nm. These two resonance bands are in fact the result of a strong coupling between the LSP and LP modes. The coupled mode with a coupling strength g results in new eigenmodes with corresponding eigenfrequencies, which can be expressed as:^[^ ^25^ ^]^

(2) $$E_{\pm }=\frac{{\tilde{E}}_{\mathrm{LSP}}+{\tilde{E}}_{\mathrm{LP}}}{2}\pm \sqrt{{\left(\frac{{\tilde{E}}_{\mathrm{LSP}}-{\tilde{E}}_{\mathrm{LP}}}{2}\right)}^{2}+g^{2}}$$

where $\tilde{E}=E-i\hbar \gamma$ in Equation (2) is a complex value. The real part *E* is the photon energy corresponding to the spectral location of the resonance, and *γ* in the imaginary part is the spectral linewidth of the resonance. Similarly, the real part of the eigenenergy corresponds to the spectral location and the imaginary part gives the linewidths of the eigenstates. When the coupling strength between the LSP and LP mode is large enough so that the minimum energy splitting between the two eigenmodes exceeds the combined spectral linewidths of the two modes, we observe an avoided crossing between the two branches of eigenmodes which is an indicator of strong coupling.^[^ ^35^ ^]^ The strongly coupled resonance modes form new quasiparticles known as polaritons and the eigenmodes are called “polariton modes,” which share similarities with the previously reported “Waveguide Plasmon Polaritons”.^[^ ^25^, ^36^ ^]^ For simplicity, we denote the lower and upper energy polaritons as *P* ^−^ and *P* ^+^ as shown in Figure 2b. The excitation condition where the spectral detuning—the spectral distance between the two original resonance modes—equals to zero, and the corresponding spectral location of the original resonance modes is the “anti‐crossing” point.

The two polariton modes asymptotically follow the two original resonance modes showing similar traits at large spectral detuning but deviating from the original resonance modes at small spectral detuning. Furthermore, the resonance mode of main influence shifts from one to another as the spectral detuning evolves from negative to positive. Based on Figure 2b, we can graphically interpret that the *P* ^−^ band is mainly influenced by the LSP mode at lower AOIs (negative spectral detuning) and then starts to show an LP mode‐like behavior at higher AOIs (positive spectral detuning), and vice versa for the *P* ^+^ band. Note that at lower AOIs we can consider the resonance mode to be purely an LSP mode instead of a polariton mode since the LP mode is non‐existent. The resonance mode emerging near 600 nm as we increase the in‐plane momentum corresponds to another localized mode that can be coupled at higher AOIs.

Near field simulation profiles of the resonance modes at different excitation conditions are given in Figure 2c, where the color coding indicates the corresponding resonances marked in Figure 2a. We see that for TE polarization, the *m* = −1 mode has a symmetric field distribution while the *m* = +1 mode has an asymmetric field. This implies that the *m* = + 1 mode is essentially an antisymmetric mode, which explains why the antisymmetric mode is only visible under oblique incidence due to its requirement of symmetry breaking. For the TM polarization, we see that the weak LP mode resonance at *θ* = 16° does not form a coupled resonance mode and therefore the near field of the supposedly *P* ^−^ band (which is yet a pure LSP mode) has a similar near field profile as the LSP mode at normal incidence. The two resonance modes couple to each other when increasing the in‐plane momentum of light, exhibiting a hybrid near field profile. As given in Figure 2d, we extracted the polariton resonance locations and performed numerical fitting to the eigenenergy functions (see Supporting Information for parameter details), which shows that the evolution of the spectral locations can be indeed explained through the strong coupling model with the distinct anti‐crossing behavior being seen.

### 2.3 Modulation Dynamics of Angular Optical Dispersion

To study the correlation between the energy evolution of hot electrons in Au and the temporal modulation of the angular optical dispersion, we utilize time and angle‐resolved pump‐probe spectroscopy. In detail, we illuminate our structure with a TE‐polarized pump light with an energy density of 9 mJ cm^−2^ that has a center wavelength of 840 nm, as schematically described in Figure 1c. The chosen pump wavelength is distant from any resonance features accessible within the studied AOI range to remove possible concerns about large inconsistency in the level of optical pumping as the incident angle changes. However, this comes at an expense of a reduced hot electron generation efficiency since a TE polarized pump cannot excite plasmon modes. Nevertheless, hot electrons are generated via the intrinsic absorption of the pump light in Au. This level of hot‐carrier‐induced refractive index tuning is sufficient to achieve a high signal‐to‐noise ratio (SNR) modulation level for characterizing the modulation dynamics. Therefore, exploring the interplay between hot electron relaxation dynamics and the optical response of our device is possible. The 840 nm pump light primarily interacts with free electrons in the conduction band and below the Fermi level *E*_F_ of gold, yielding energetic carriers with a nonthermal energy range of *E*_F_ < *E* < *E*_F_ + 1.48 eV, which quickly thermalizes through electron‐electron scattering. The thermalized electrons now have an elevated effective electron temperature higher than that of the room temperature. Such procedure results in the modulation of the Au relative permittivity, and subsequently the optical interaction of the probe beam with the plasmonic structure. Note that the selection of TE polarization further offers the opportunity to minimize hot electron injection from the plasmonic structures to the nearby dielectrics which, if present, results in ultrafast processes governed by different relaxation pathways.

**Figure 3:**
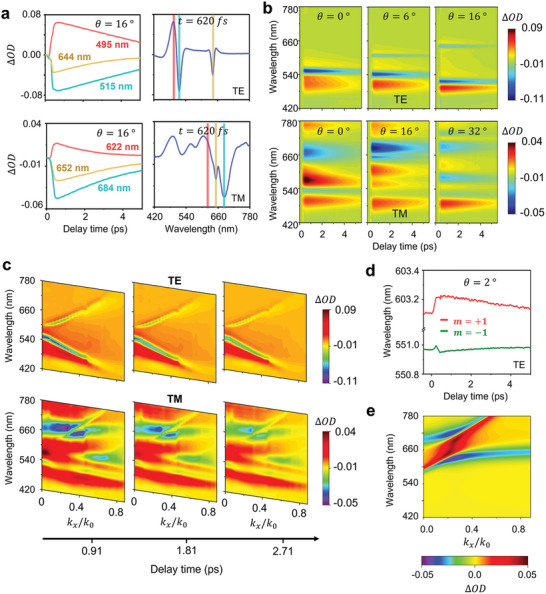
Transient response of the optical dispersion relation of the plasmonic structure. a) 1D graphs of the transient optical response of the nanograting. The left panels show the time evolution of the ΔOD values at selected wavelengths, and the right panels show the dispersion of the modulation depth at a given timestamp where the modulation amplitude is close to maximum. The color bars in the right panels correspond to the spectral location of the selected wavelengths from the left panel. b) 2D maps of the transient optical response of the nanograting under varying AOIs, which indirectly indicate the spectral locations of the optical resonances. c) 2D maps of the transient angular dispersion at different time stamps for TE and TM probes. Resonance bands of both the optical resonances and the angle‐independent material response (which originates from the modified interband transition rate) are apparent in the 2D maps. d) Spectral location of the two resonance bands at an AOI of 2°. An increased band separation can be seen following the pump excitation. e) The characteristic features in the measured results using the TM probe reproduced with a simplified qualitative model. A spectral broadening of the LSP mode results in the traits of the transient angular dispersion observed near the anti‐crossing point.

A representative transient response of the sample at *θ* = 16° is given in **Figure** **3**a. The change in the optical response of the sample is depicted by the change in the optical density (OD), which is calculated as ΔOD = −log (*I*′/*I*_0_). *I*_0_ is the static intensity of the transmitted probe light, and *I*′ is the modulated intensity at specified time instances after the arrival of the pump pulse. The left panels of Figure 3a show the time evolution of ΔOD at various wavelengths under both polarization states at a representative AOI of *θ* = 16°. Furthermore, by plotting ΔOD as a function of wavelengths at a given time stamp (as we see in the right panels of Figure 3a), we can understand how the optical resonance modes of the sample respond to the refractive index change. Expanding the ΔOD − *λ* plot in the time domain gives us the 2D plots presented in Figure 3b. Although the 2D maps help us track the resonance dependent transient response, some of the features are not easy to interpret within the given configuration. This motivates us to plot the angular dispersion of the optical response modulation at different time stamps.

The angular dispersion maps of the transient response are presented in Figure 3c, where the resonance band structures are shown with remarkable clarity compared to the static optical dispersion maps in Figure 2b accompanied by spectral shifts particularly for the plasmonic modes. The transient response under TE‐polarized illumination is depicted in the upper half of Figure 3c. Here we observe that the GMR band of the upper antisymmetric mode spectrally red shifts while the lower symmetric mode band blue shifts, resulting in an increased energy gap between the two bands. This can be qualitatively explained through Equation (1), where the spectral separation between the upper and lower GMR bands is proportional to *n*_eff_. Indeed, the photoexcitation of hot electrons transiently increases the real part of the refractive index of gold, which subsequently increases *n*_eff_ as well and as a result widens the energy gap between the two GMR modes. As provided in Figure 3d, we observe the resonance locations moving in opposite directions for the exemplary case of an AOI of 2°. Note that the major portion of the red colored region near the lower band at small AOIs originates from an increase in material absorption due to enhanced interband electronic transitions in gold, and therefore only a moderate shift in the resonance location is observed.

The transient maps at the lower half of Figure 3c also reveal the impact of hot‐electron generation on the dispersive optical interaction of a TM‐polarized light with the sample. First, regardless of the in‐plane momentum of light we see modulation features near a 500 nm wavelength that stem from the modified interband transition rate of electrons. This occurs upon the perturbed distribution of electrons in the sp‐band energy states around the Fermi level, following the absorption of 840 nm pump photons. In addition, near the spectral regions corresponding to the LSP resonance (e.g., 680 nm at the normal incidence), the differential transmission response shows a negative value that is surrounded by two positive spectral features. Such a transient response indicates a spectral broadening of the plasmon resonance accompanied by a subtle spectral shift due to the excitation of hot electrons, which is consistent with previous reports.^[^ ^37^, ^38^ ^]^ The dominant role of resonance broadening originates from the large hot‐electron induced absorption loss, which has a larger impact on the overall modulation behavior of the device compared to induced resonance shifts. This trait results in the distinct relative modulation depth observed as purple‐colored spots in the transient 2D maps, near the anti‐crossing point seen around 660 nm and *k_x_*/*k*_0_ = 0.3.

Under the dominance of spectral broadening, the modulation depth is proportional to the ratio between the perturbed linewidth and unperturbed linewidth of the polariton bands. The ratio can be maximized by increasing the amount of spectral broadening and minimizing the linewidth of the static response. The linewidth broadening of the LSP mode is much larger compared to the LP mode in terms of the absolute change, and therefore polariton modes exhibit a larger spectral broadening at regions where the mixing fraction of the LSP is larger. In contrast, the minimization of the unperturbed linewidth requires the LP mode to have a larger influence on the polariton bands. Considering the influence shift between the two original resonances within the polariton modes, there is a tradeoff relation between a smaller polariton mode linewidth (influenced by the LP mode) and larger spectral broadening (due to the LSP mode). Therefore, near the anti‐crossing point is where the tradeoff between the static LP and transient LSP mode can be optimized and therefore achieve larger modulation. To verify this qualitative explanation, we analytically reproduce the results using a simplified qualitative model. We first mimic the transmission spectrum around the long wavelength range by employing two Lorentzian curves to model the polariton modes our structure supports. The spectral locations and linewidths following Equation (2) are given as the input for the two Lorentzian functions, where the properties of the original resonance modes are manually defined. We then calculate the new transmission spectra after increasing the spectral linewidth of the low‐Q resonance mode which comprises the coupled oscillation. The resulting modulation is graphically presented as shown in Figure 3e, fairly reproducing the major features of the measured transient angular dispersion, under a TM polarized illumination. Despite the discrepancies that we observe, the model successfully reproduces key features to support our qualitative understanding.

### 2.4 Temporal Dynamics of the Angular Dispersion

**Figure 4:**
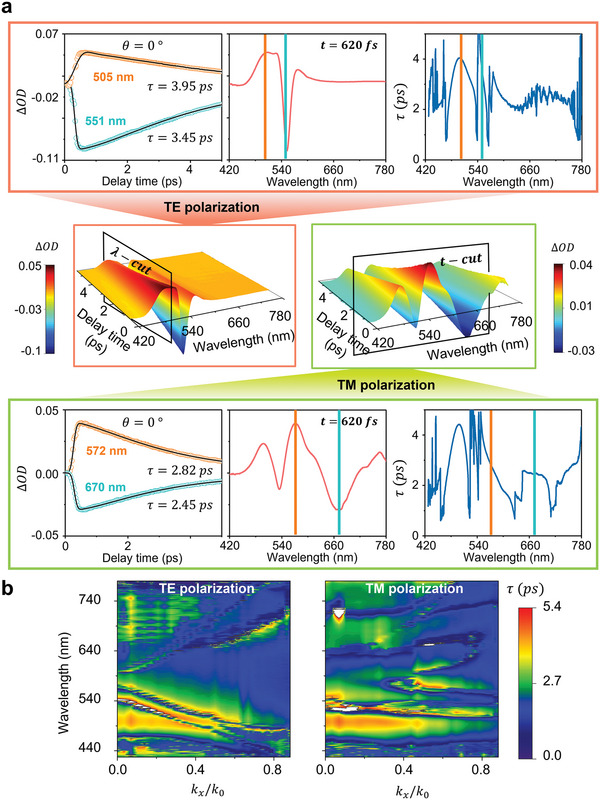
Angular dispersion of the recovery time constant. a) A graphical introduction to the dispersion of the recovery time constant. A 3D representation of the transient 2D map at normal incidence is given at the central region of the figure, with the cut directions indicated. The cross‐sectional views of the 3D plots with similar cuts are given in the upper and lower boxes for different polarizations. The left panels in the boxes depict the time evolution of the transient optical response at selected wavelengths. Analytical functions obtained through numerical fitting are plotted together on the time evolution graph with the recovery time constant values provided. The middle panels show the ΔOD − *λ* graphs, which represent the dispersion of the modulation depth. The dispersive property of the temporal behavior can be presented similarly by plotting the *τ* − *λ* curves shown in the right panels. The color bars in the ΔOD − *λ* and *τ* − *λ* graphs represent the spectral location of the selected wavelengths in the left panels, showing the relative spectral location within the optical resonances and their corresponding time constants. It is worth mentioning that a correlation between ΔOD − *λ* and *τ* − *λ* is noticeable. b) 2D maps of the angular dispersion of recovery time constants for TE and TM polarization. Remarkably, resonance bands are yet again observed showing characteristic features of spectral broadening. The slow dynamics near the interband transition of gold is observed for both polarizations in a form of large time constant bands, where a modification due to the photonic resonance mode is observed for TE polarization. The observation implies that the recovery time is a result of the interplay between the carrier relaxation dynamics and the optical resonances.

The tuning of resonance properties via the control of the in‐plane momentum of light allows for the exploration of the dispersive temporal dynamics of the light interaction with our hybrid platform in the presence of spectrally tunable resonance modes. In order to analyze the temporal dynamics, we numerically fit the transient dynamics of the optical response at all measured wavelengths and under all explored AOIs using the summation of three exGaussian functions (see Supporting Information for details). Following the fitting, we construct a set of 2D maps in which the retrieved time constants, *τ*, are plotted as a function of the wavelength and normalized in‐plane momentum of the probe light (i.e., *τ*(*λ*, *k_x_*/*k*_0_)). Note that all 2D maps are constructed based on the slowest finite time constant that describes the decay of ΔOD at all probe wavelengths. Some exemplary results for the fitting process (solid black lines) are provided in the left panel within the upper and lower boxes of **Figure** **4**a, revealing a perfect match with the measured data (circles). When comparing the ΔOD(*λ*) and *τ*(*λ*) plots, there is a notable correlation between the recovery time and the dispersive optical response of the structure, generally witnessing a slower relaxation process as we approach various types of resonance modes. Macroscopically, the nonlinear spectral profile of the resonance modes serves as the origin of temporal dispersion. The modulation rate is indeed a nonlinear function of linewidth perturbation, spectral shift, and wavelength, which produces the dispersion of decay time deviating from hot electron lifetime.

Figure 4b shows *τ*(*λ*, *k_x_*/*k*_0_) plot under both TE‐ and TM‐polarized illuminations. The intriguing observation is that the dispersive nature of the light transmission through the sample is fully projected into the extracted relaxation time constant. By taking a closer look, one can notice that at the spectral vicinity of the photonic and plasmonic resonance bands we have a slow recovery process (i.e., large *τ*). This is surrounded by notably faster relaxation time constants, which then recover back to an intermediate level as we spectrally move further away from the resonance bands. To understand the origin of such dispersive temporal dynamics we examine the impact of the hot‐electron generation on the real and imaginary part of the refractive index change, as hot electrons journey through the thermalization processes. The formation of hot carriers in gold primarily perturbs the distribution of electrons in the energy band, which enhances the interband and intraband electronic transition rates owing to the depopulation of a wide range of energy levels below *E*_F_. The enhanced transition rates can be collectively described via an increase in the effective imaginary part of the gold refractive index. In addition, upon the emergence of the formation of a thermalized hot‐electron system, the elevation of the effective electron temperature leads to further modulation of the Au indices of refraction, in particular its real part.^[^ ^2^ ^]^ The resonance wavelength and bandwidth of a mode are primarily impacted by the real and imaginary parts of the refractive index, respectively. Hence, the observed modulation behaviors distant from a resonance mode mostly stem from the spectral broadening due to the hot‐electron induced loss, while at the resonance wavelength both spectral broadening and shift contribute to the modulation of light transmission through the sample. Combined with the induced temporal dispersion due to intrinsic nonlinearities of optical resonances, at wavelengths away from the center of resonance, we observe smaller recovery time due to the faster relaxation of the imaginary part of the refractive index over the real part. Furthermore, the existence of exceptional switching speeds coinciding with the nodal points, which are zero‐crossing points observed in ΔOD(*λ*) plots, stems from the nonlinear response near the spectral region with respect to the change in the refractive index (see Supporting Information for detailed discussion). In general, the observation implies that optical resonances can both expedite or retard the modulation speed compared to the hot‐electron lifetime at certain spectral regions. The singularity points, including the white features in Figure 4b which are diverging relaxation time constants near the nodal points of the ΔOD spectra, are likely data processing artifacts due to bad SNRs and transient behaviors differing from other spectral regions. Our observation in a relatively broad wavelength range is expected to be entangled with the modulation response of any all‐optical switch based on a carrier‐driven nonlinear effect rather than a purely coherent nonlinear interaction.^[^ ^2^ ^]^

It is also worth mentioning the large time constants forming around the 500 nm wavelength, which is near the onset energy of the d‐band transition of gold. Due to the long‐lasting energetic electrons near the Fermi level, the carrier dynamics regarding the interband transition is generally slower than the intraband transition. Therefore, the polariton modes and the upper band of the photonic mode show a faster response compared to the intrinsic material response near 500 nm. However, in the TE polarization map, we also see that the previously mentioned temporal characteristics of resonance modes shadow the slow material response near 500 nm and allow us to achieve a faster recovery time. The observed possibility is intriguing once we notice that the large Kerr‐type optical nonlinearity of Au has been often regarded as a slow route for implementing an optical switch. This again emphasizes the role of resonance modes in the dispersion of recovery time, suggesting that the recovery time can be controlled through optical engineering of resonance modes to enter a regime of speed that exceeds limitations imposed by intrinsic material responses. Moreover, the recovery time at a certain wavelength can be simply controlled by the in‐plane momentum of light which gives post‐fabrication tunability to the optical device performance. The gradual decay of the time constant near 500 nm as we increase the in‐plane momentum is due to the decrease in the pump effective fluence on the surface of the sample. Since we kept the pump power constant while changing the AOI, the beam size increases, which reduces the pump fluence. The power dependence of the recovery time is well‐known,^[^ ^39^ ^]^ and could be a good indicator of excitation power level when we map the time constants.

## 3 Conclusion

An in‐depth study unraveling the interplay between carrier dynamics and the optical resonance was presented by investigating the angular dispersion of the transient response of a hybrid photonic‐plasmonic crystal. The integration of a plasmonic nanograting with a dielectric slab allows for enabling photonic and plasmonic modes in a spectrally tunable fashion by leveraging the in‐plane momentum of light into the devised hybrid platform. This configuration thus grants the capability to support efficient optical switching throughout nearly the entire visible regime. We then performed angle‐resolved transient pump‐probe spectroscopy and extracted the angular dispersion of transmission in the time domain. As part of the angle‐resolved all‐optical switching, coherent control of the PBG was demonstrated for the photonic resonance modes, and enhanced modulation sensitivities near the anti‐crossing point were revealed for strongly coupled plasmonic resonance modes. The observed large modulation near the anti‐crossing points was primarily attributed to the imbalance between the linewidths of the two interacting resonance modes and the amplitude of spectral broadening taking place. Moreover, the measured 2D map of the recovery time constant showed the impact of hot‐carrier dynamics entangled with engineered photonic and plasmonic resonances on the modulation speed. The results suggest that a specific recovery time at a desired spectral location could be achieved through tailoring photonic and plasmonic resonances and manipulating the incident momentum of light within the plane of devised platform. Thus, the photonic resonance near the interband transition of Au offers broader choice of modulation specifications, overcoming material limitations in terms of both resonance strength and modulation speed. In reverse, this implies that probe wavelengths should be carefully selected if hot carrier lifetimes are to be measured through optical means since pre‐existing optical resonances may alter the recovery time of modulated optical signals upon hot‐carrier excitation. Our findings thus provide fundamental insights for studying hot‐carrier dynamics and designing plasmonic nanostructure platforms for all‐optical control exploiting hot carriers, bringing us one step closer to utilizing hot carriers at their full potential.

## 4 Experimental Section

### Device Fabrication

The sample fabrication process begins with depositing a 25 nm‐thick TiO_2_ layer on a glass substrate (Corning, C1737 glass) with plasma assisted atomic layer deposition at 250 °C using a Cambridge Fiji Plasma ALD system. Following the film deposition, the nanograting was formed on top of the TiO_2_ film in a three‐step fabrication process: i) a standard e‐beam lithography process (Elionix ELS G‐100) to define the nanopatterns, ii) e‐beam evaporation of 2 nm/45 nm Ti/Au metal using Denton Explorer, and iii) a lift‐off process in acetone to resolve the plasmonic structures. The nanograting was covered with an additional 45 nm‐thick TiO_2_ layer through ALD under the same condition.

### Optical Characterization

The ultrafast pump‐probe spectroscopy setup was operated through a regenerative amplified Ti:sapphire femtosecond laser system (Spectra‐Physics Solstice Spitfire ACE, 2.5 kHz repetition rate, 89.3 fs pulse width, 800 nm wavelength, and a pulse energy of 1.6 mJ per pulse), and the data collection was conducted using a Helios spectrometer (Ultrafast Systems Inc.). An 80:20 beam splitter divides the 800 nm fundamental light from the laser system into two beams. The low intensity part of the fundamental light passes through another 80:20 beam splitter, and the power level gets reduced again by 80%. The laser beam then gets focused on a 2 mm thick sapphire window to generate a white light continuum (WLC) as the probe signal. Prior to WLC generation, the low intensity beam first passes through a motorized delay stage in order control the delay between the pump and probe signal, which has a minimum resolution of 30 fs. The probe beam was then focused onto the sample using a parabolic mirror, and the transmitted probe light was collected by an optical fiber coupled into a visible spectrometer. The high intensity part was used to operate the optical parametric amplifier (Ultrafast Systems Inc. Apollo). The fundamental light was partially converted into two near‐IR pulses, signal and idler, and the combination between the three beams and different nonlinear processes produces single wavelength pump pulses with a desired wavelength. The pump pulse was also focused on the sample through a parabolic mirror, where the pump was incident with a 10° offset from the probe light. For angle‐resolved measurements, the sample was mounted on a rotation stage attached to a linear stage (Thorlabs). The linear stage was used to locate the sample at the focal point of the pump and probe, and the rotation mount was used to change the AOI by rotating the sample. An instrument response function value of 150 fs typically returns good numerical fittings for the transient kinetics, and therefore it was safe to consider that the setup was suitable to detect ultrafast processes with a timescale slower than 150 fs. Chirp corrections and coherent artifact removal of the obtained transient maps were done via post processing.

## Conflict of Interest

The authors declare no conflict of interest.

## Supporting information

Supporting Information

## Data Availability

The data that support the findings of this study are available from the corresponding author upon reasonable request.
