# Supplementary material for: Tailored Dispersion of Spectro‐Temporal Dynamics in Hot‐Carrier Plasmonics
Source: Adv Sci (Weinh). 2023 Jan 19;10(8):2205434. doi: 10.1002/advs.202205434 (PMC10015883; doi:10.1002/advs.202205434)
Supplement: Supplementary file 1 — Supporting Information [file ADVS-10-2205434-s001.pdf]

Supporting Information for

Tailored Dispersion of Spectro-Temporal Dynamics in Hot-Carrier Plasmonics

Andrew S. Kim[1‡], *Mohammad Taghinejad*[1‡], *Anjan Goswami*[1], *Lakshmi Raju*[1], *Kyu-Tae Lee*[1] *and Wenshan Cai*[1,2*]

[1] School of Electrical and Computer Engineering, Georgia Institute of Technology, Atlanta, Georgia 30332, USA

[2] School of Materials Science and Engineering, Georgia Institute of Technology, Atlanta, Georgia 30332, USA

[‡] These authors contributed equally to this work.

*E-mail: wcai@gatech.edu

1. Static optical properties of the jagged region in the lower energy band of GMR

Figure 2b in the main text, which is the simulation results for the angular dispersion of optical transmission under TE illumination, shows distinct jagged features near the excitation condition of $k_x/k_0 = 0.3$ and $\lambda = 630\ nm$. High-resolution simulation near the region is given in Figure S1a, where the step size for the AOI was decreased from 2 ° to 0.2 °. Here we no longer see the jagged patterns indicating that such feature is simply an artifact of the lack of spectral and angular resolution of the simulation. This feature is observed due to the spectral linewidth of the resonance mode being much narrower compared to the magnitude of the spectral shift which can be seen in Figure S1b. The reflection and absorption maps are also plotted where we see a mismatch in the spectral and angular regions for maximum reflection and absorption. The results are shown in Figure S1c and Figure S1d respectively.

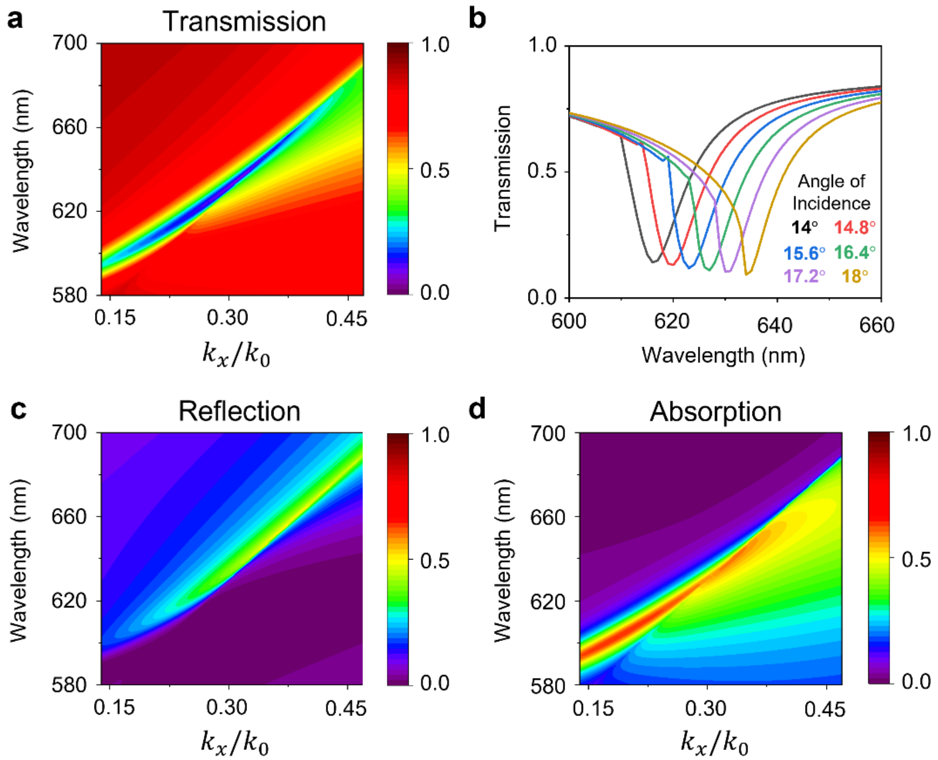

Figure S1. a) Simulated 2D angular dispersion map of the transmission response with denser interval. b) Transmission curves at selected AOIs to show the fast spectral shift compared to the linewidths. c-d) Simulated 2D angular dispersion map with denser interval for reflection and absorption, respectively.

2. Distinguishing between lattice plasmon resonance and guided mode resonance

Both lattice plasmon (LP) mode and guided mode resonance (GMR) occur due to diffractive coupling and thus the two modes show similar spectral behavior, where the resonance locations are sensitive to the in-plane momentum of the impinging light. This makes it often confusing to properly identify whether a resonance mode is either a LP mode or a GMR under TM-polarized illumination. We deduce that the resonance dependent to the angle of incidence (AOI) that we observe under TM incidence is indeed an LP mode based on two reasons. First, the spectral locations of the high-Q resonance beyond 800 nm are well above the cut-off wavelength for TM waveguide mode in the current configuration. Second, the resonance dip at lower AOIs vanishes since LP mode is sensitive to the plasmonic response of the resonators and requires a strong out-of-plane dipole coupling.[1] Therefore, both the dampened plasmonic response at shorter wavelengths and small AOIs make the LP mode disappear, where the resonance should have been observed if the resonance originated from GMR. This also explains why we only see one resonance dip instead of two for the LP mode resonance at oblique incidence.

3. Parameters for the coupled oscillator model

The analytical expression of the eigenfrequencies of the two polariton modes that describes the experimental results most accurately was obtained by fitting Eq. (2) in the main text to the experimental values given in Figure 2d of the main text. The spectral location of the uncoupled LSPR mode was fitted as a constant, which its location lies within the spectrally broad resonance mode near 720 nm. The spectral location of the uncoupled SLR mode was fitted to the following equation: [2-3]

$$\lambda_{SLR} = p \cdot n_{\text{eff}} \cdot (1 + \sin\theta) \qquad \text{(S1)}$$

Here, p is the periodicity of the devised structure, n_{eff} is the refractive index of the surrounding environment of the plasmonic gratings, and θ is the angle of incidence. The spectral locations described in wavelengths were then converted into photon energy expressions. Numerical fitting was executed via MATLAB where the fminsearch algorithm was implemented.

Table S1 List of parameter values obtained through numerical fitting of the coupled oscillator model to the experimental results.

**Parameter**	**Value**
$\boldsymbol{\lambda_{LSP}}$	723 nm
$\boldsymbol{n}_{\mathbf{eff}}$	1.414
**g**	68.5 meV

4. Absorption variation upon varying angle of incidence

In order to accurately analyze the switching dynamics measured through transient pump-probe spectroscopy on the optical transmission, a rigorous understanding of the excitation condition is required, where maximum electron temperature and thus the modulation depth and recovery speed depends on the absorbed power incident on the sample. Considering that the change in the excitation condition upon sample rotation couldn't be avoided, we need a clear picture regarding how the absorption and the incident intensity of the pump light varies as a function of the AOI. The situation however is more complicated than it seems due to the oblique incidence of the pump light relative to the probe light. Looking at Figure S2 where angle notations are graphically described, at the excitation condition with zero sample rotation the pump light has an angle of incidence (φ) of 10° in the y-z plane which can be understood as the azimuthal angle (α) being 90°. As we increase the AOI (θ) for the probe light through sample rotation, both the AOI (β) and azimuthal angle (α) of the pump light changes. The relationship between the angles α, β, θ, and φ can be described through the following equations:

$$\cos\beta = \cos\varphi \cdot \cos\theta \qquad \text{(S2)}$$

$$\cot\alpha = \frac{\cos\varphi \cdot \sin\theta}{\sin\varphi} \qquad \text{(S3)}$$

The absorption of the nanograting structures at 840 nm can be simulated through COMSOL with the given angle of incidences and azimuthal angles. Given that the incident power level was kept constant, changing the AOI also modifies the intensity of the pump light due to varying cross sectional area of the laser beam where the intensity change is proportional to $\cos\theta$. Thus, the effective absorptance upon varying AOIs can be computed as:

$$A_{\text{eff}}(\theta) = P_{\text{abs}}(\theta)/P_0 \ = A(\theta) \cdot \cos\beta(\theta) = A(\theta) \cdot \cos\varphi \cdot \cos\theta \quad \text{(S4)}$$

where P_0 is the pump power level at normal incidence. The numerical results of the absorbed power as a function of the sample rotation (θ) is shown in Figure S3.

Due to the initial angular offset, the polarization direction of the pump light relative to the grating also changes upon sample rotation. Following the coordinate conventions of Figure 1a in the main text, the polarization rotation of the TE and TM polarizations as a function of varying AOIs can be expressed as:

$$\text{TE:} \ -\sin\varphi \cdot \sin\theta\, \hat{x} + \cos\varphi\, \hat{y} - \sin\varphi \cdot \cos\theta \ \hat{z} \quad \text{(S5)}$$

$$\text{TM:} \ \cos\theta \ \hat{x} - \sin\theta \ \hat{z} \quad \text{(S6)}$$

Given that the angular offset of the pump light is 10°, the major portion of the incident power remains in the y-component of the electric field which is parallel to the grating structure regardless of the angle of incidence. Therefore, the variation in the excitation condition due to polarization rotation can be neglected.

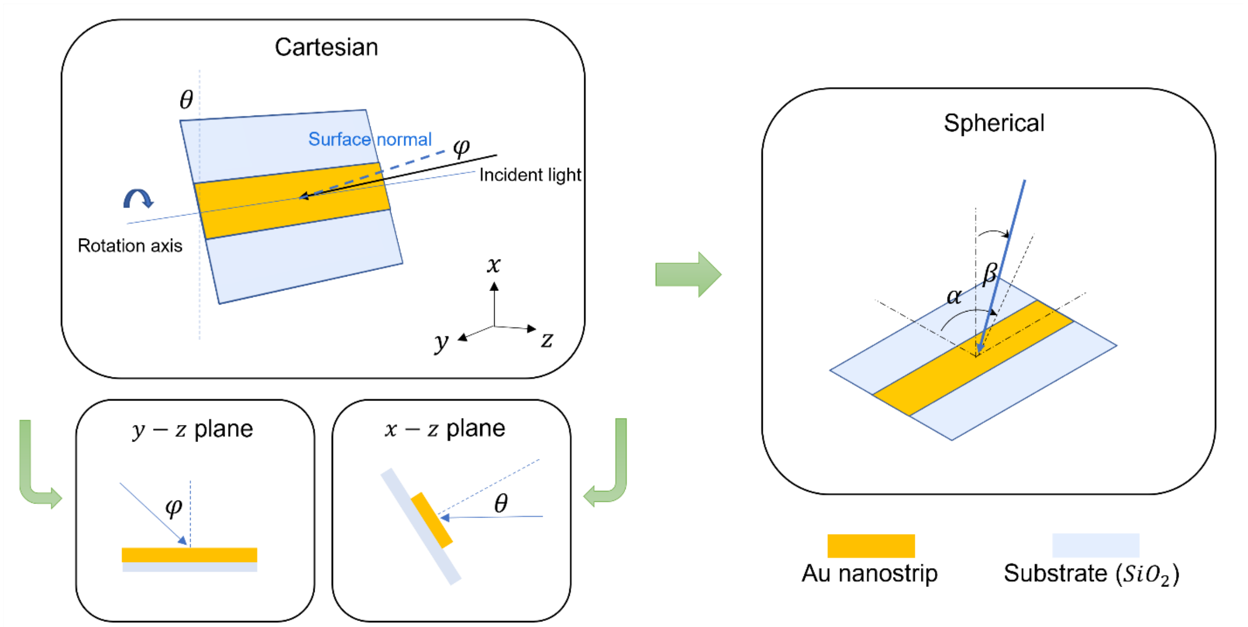

Figure S2. Notation conventions of different angles defined in cartesian and spherical coordinates. The cartesian coordinates reflects the employed experimental setup which needs to be converted into spherical coordinate conventions for COMSOL simulation.

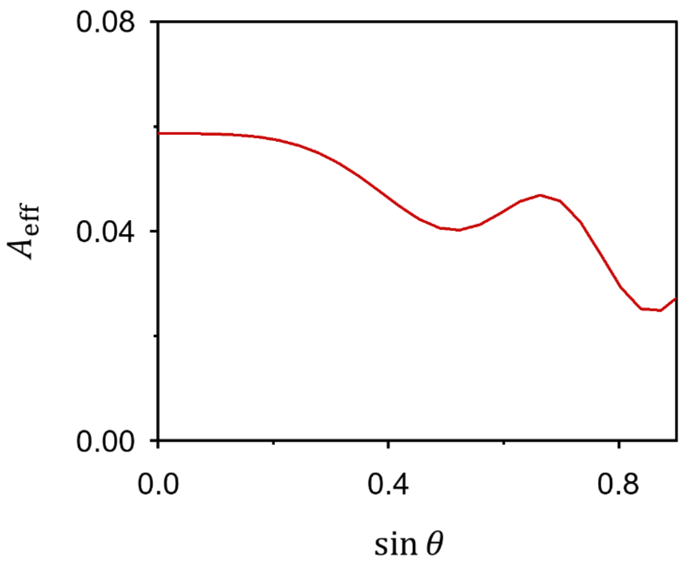

Figure S3. Graph of the effective absorption as a function of $\sin\theta$ calculated through numerical simulations, where θ is the sample rotation angle.

5. Contribution of nondegenerate two-photon absorption to the transient response

In a two beam experiment configuration, simultaneous excitation by overlapping pump and probe signal can lead to non-degenerate two-photon absorption (TPA), which can contribute to the transient response if the effect is strong enough. In order to elucidate the influence of the nonlinear effect in our experimental results, a transient pump-probe measurement was repeated with fixed pump conditions and varying probe pulse power. The pump condition was identical to the described conditions in the main text and was probed with TE polarized light at normal incidence. The probe pulse powers are denoted in terms of powers relative to the maximum achievable power level that doesn't saturate the detection signals from the photodetector. The results show nearly identical transient response, where a representative modulation curve at a selected wavelength is presented in Figure S4. A monotonic trend upon increasing probe intensity cannot be seen and therefore the influence of non-degenerate two-photon absorption to the transient response can be ruled out in our experimental results.

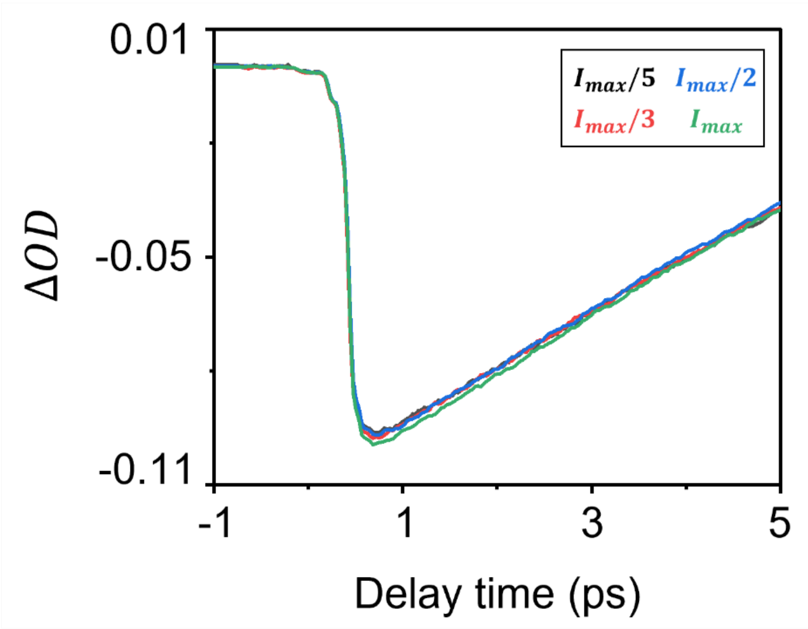

Figure S4 Transient response curves with varying probe intensities at $\lambda = 544\ nm$. I_{max} denotes the maximum probe intensity acheivable before the onset of saturation of the photodetector. The probing condition was fixed to TE polarization at normal incidence.

6. Simplified qualitative model for polariton modulation analysis

We first start with defining the linewidths and resonance wavelengths of the two uncoupled resonances at different AOIs. The linewidth for the LSP mode is retrieved at normal incidence, and the linewidth for the LP mode is retrieved at high AOIs where the perturbation due to coupling is small. We then compute the resonance locations and linewidths of the two polariton modes under all AOIs based on eq. (2) in the main text. The transmission spectra each resulting from the polaritons are assumed as Lorentzian curves, and the overall transmission spectra was obtained by averaging the contributions from the two polariton curves for simplicity. We now repeat the process however with a perturbed linewidth for the LSP mode. Having obtained the angular dispersion of the perturbed and unperturbed transmission of the 1D crystal, the change in optical density can once again be calculated as $\Delta OD = -\log(T'/T_0)$, which gives the results we see in Figure 3e. Some exemplary curves visually describing the modelling process is given in Figure S5.

As discussed in the main text, Figure S6 shows that coupled oscillation modes formed from resonances with similar linewidths will not result in maximum modulation depths near the anti-crossing point. Here, we assigned linewidths of LP mode resonances to be the same as the LSP mode, and repeated the entire process described above. This further confirms our qualitative analysis, since there are no such trade-off relations between the static linewidth and the perturbed linewidth.

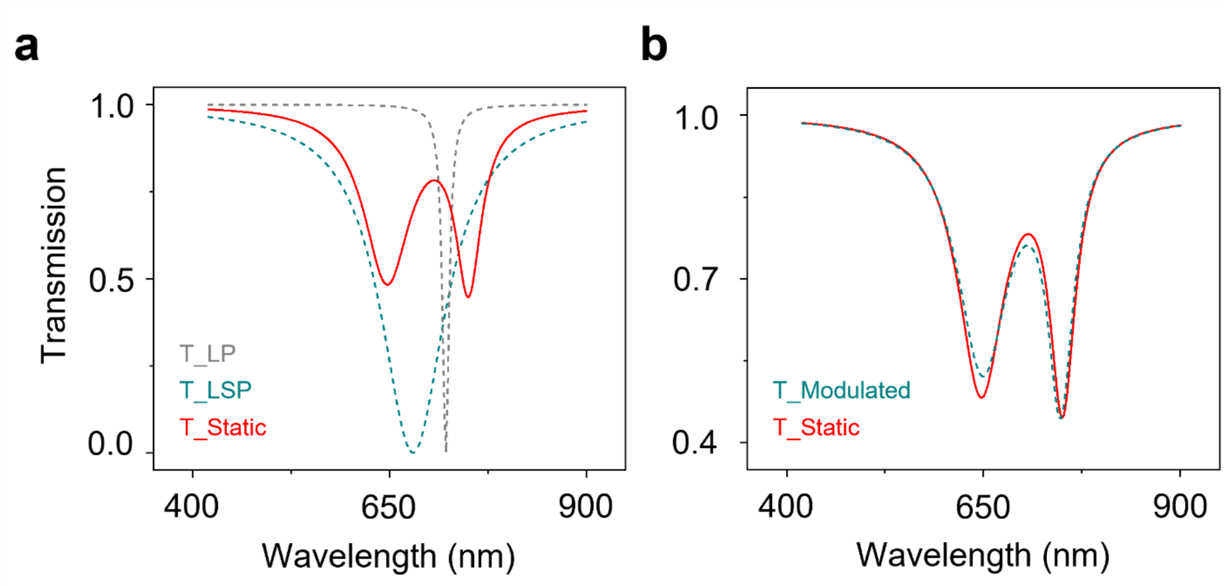

Figure S5. a) Transmission graphs depicting the formulation of the simplified qualitative model. Two Lorentzian curves representing the uncoupled resonance modes (LP and LSP) are given, where the transmission curve corresponding to the resulting coupled resonance (Static) is graphed together. The difference in the depth of the resonance dips occur since the static transmittance is defined as the mean value of the newly obtained Lorentzian curves representing the Polariton modes. b) Comparison between the spectral profile of the static and modulated transmission curves. The modulated curve is obtained by changing the linewidth of the LSP resonance that comprises the coupled resonance mode. Note that the modulation is purposely exaggerated to make the deviation look more obvious.

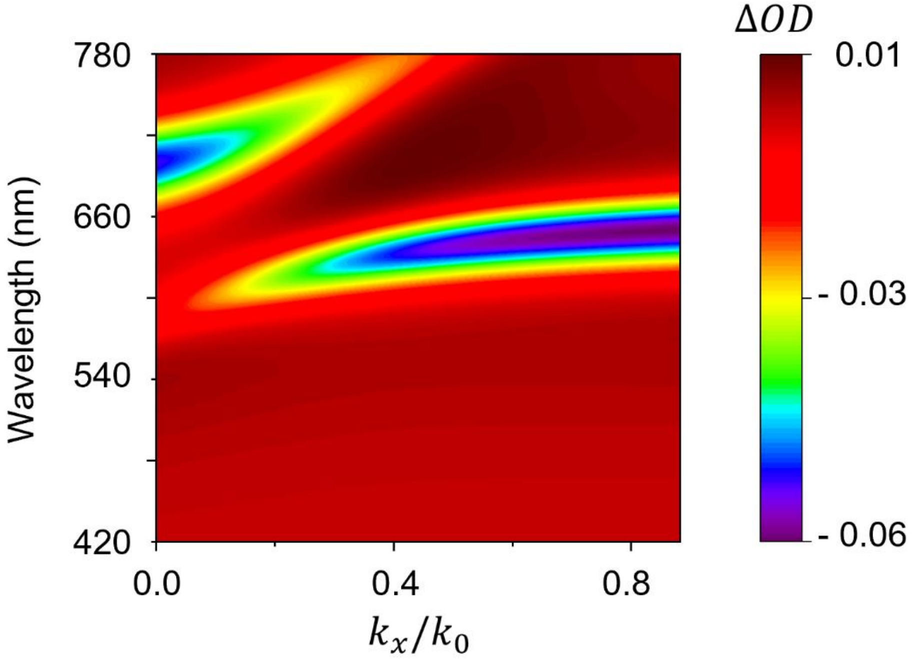

Figure S6. Differential angular dispersion map produced with two fundamental resonances with same linewidths. A large modulation is observed at regions with large LSP resonance mixing fraction within the polariton branches since the curve equivalent to LSP resonance was only perturbed. Since there are no tradeoff relations as discussed in the main text, we don't see any modulation enhancement near the anti-crossing point in this case.

7. Estimation of the induced refractive index change within the plasmonic structures

A widely employed approach for analytically modeling the ultrafast response of a plasmonic structure consists of two steps. First, model the change in the interband transition rate and intraband transition rate of the plasmonic material as a function of the population of non-thermal electrons and the effective electron and lattice temperatures. Second, through electromagnetic simulation, compute the differential response of the plasmonic nanostructures given a minuscule perturbation in the refractive indices (or the relative permittivity). Combining the previous steps, the overall change in the linear transmission upon refractive index modulation can be expressed as:

$$\Delta T = \left.\frac{\partial T}{\partial \varepsilon'}\right|_{\varepsilon' + i\varepsilon''} \Delta\varepsilon' + \left.\frac{\partial T}{\partial \varepsilon''}\right|_{\varepsilon' + i\varepsilon''} \Delta\varepsilon'' \quad \text{(S7)}$$

$$n = Re(\sqrt{\varepsilon' + i\varepsilon''}) \quad \text{(S8)}$$

$$k = Im(\sqrt{\varepsilon' + i\varepsilon''}) \quad \text{(S9)}$$

As the reference for the estimation of refractive index change, we choose the transient response of our structure when probed by TE polarized probe at normal incidence. The linearized coefficients for induced relative permittivity change of gold (Au) as a function of effective electron temperature was retrieved from literature.[4] Given that major features of the transient response are located near 552nm, we neglect the contribution of the change in the intraband transition rate which is present in longer wavelengths. The modeled differential response is given in Figure S7a, and the subsequent refractive index change is plotted as a function of wavelength which is shown in Figure S7b. In the figures we see that the modeled differential response shows good agreement to our experimental results shown in Figure 4a in the main text, which implies

that our estimation of the induced change in refractive index is indeed within a reasonable range compared to the actual value. Here, the discrepancy in the linear optical transmission predicted by the numerical simulation and the measured optical response is propagated throughout the differential response modelling, which can be observed as a slight blueshift of the modulation features numerically predicted compared to the experimental values.

Although beyond the scope of our work, the accuracy of the estimations can be further improved by incorporating the inhomogeneous three-temperature model (I3TM) and considering the contribution from the change in the intraband transition rate of Au for refractive index modelling.[5] Furthermore, instead of the linear approximation for the differential response, we can simulate the linear response of the structure with refractive indices at different time stamps. This approach aligns better with our work given that the linearization assumes that the switching speed is identical throughout the entire wavelength and strictly follows the carrier relaxation time which is contrary to this study.

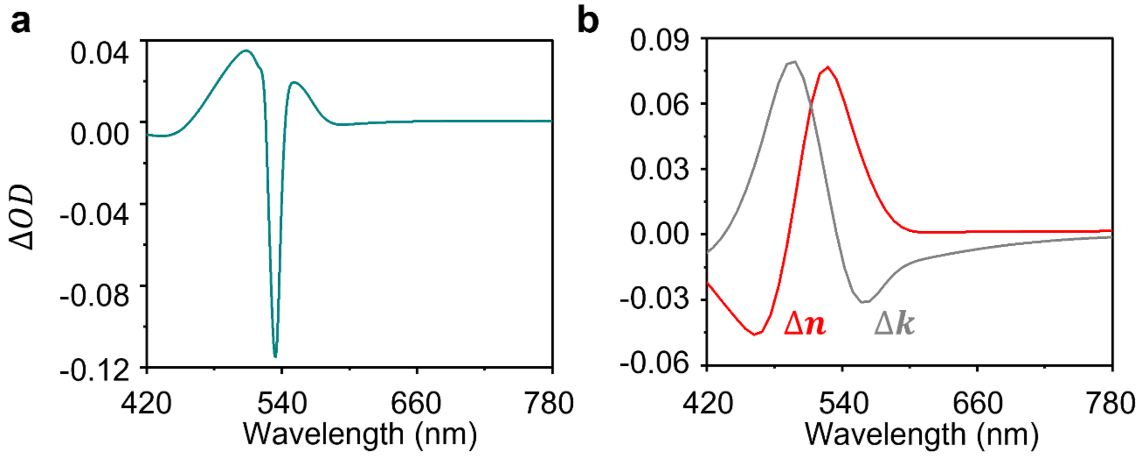

Figure S7 a) Calculated transient optical response of the sample at maximum modulation depth b) Corresponding change in the real and imaginary part of the refractive indices of Au

8. Numerical retrieval of the time constants

The time constants of each and every wavelength and in-plane momentum of probe light was retrieved by fitting the corresponding transient optical response of the Au nanograting at the given condition using MATLAB. The transient optical response was fitted to a function known as the ex-gaussian function which results from the convolution between a gaussian pulse and a exponential decay function. Three ex-gaussian functions with different time constants each corresponding to the rise, recover, and phonon-phonon scattering process were used to describe the transient optical response. Note that in our case the phonon-phonon scattering contribution was accounted as a constant since the timescale is much larger compared to the electron-electron and electron-phonon scattering associated rise and recovery time. The resulting analytical form of the fitted transient optical response (ΔOD) is given as:

$$\Delta OD_{\lambda,k_x}^{\mathrm{fit}}(t) = A_{\lambda,k_x}^{\mathrm{rise}} \cdot e^{-\frac{t-t_0}{\tau_{\lambda,k_x}^{\mathrm{rise}}}+\left(\frac{t_p}{2\tau_{\lambda,k_x}^{\mathrm{rise}}}\right)^2} \cdot \left[1+\mathrm{erf}\left(\frac{t-t_0}{t_p}-\frac{t_p}{2\tau_{\lambda,k_x}^{\mathrm{rise}}}\right)\right]+\cdots$$

$$+\ A_{\lambda,k_x}^{\mathrm{recover}} \cdot e^{-\frac{t-t_0}{\tau_{\lambda,k_x}^{\mathrm{recover}}}+\left(\frac{t_p}{2\tau_{\lambda,k_x}^{\mathrm{recover}}}\right)^2} \cdot \left[1+\mathrm{erf}\left(\frac{t-t_0}{t_p}-\frac{t_p}{2\tau_{\lambda,k_x}^{\mathrm{recover}}}\right)\right]+\cdots$$

$$+\,A_{\lambda,k_x}^{\infty} \cdot \left[1+\mathrm{erf}\left(\frac{t-t_0}{t_p}\right)\right] \qquad \text{(S10)}$$

t_0 in the equation stands for the time zero of the transient response, $t_p = IRF/2\ln 2$ where IRF stands for instrument response function which the value was fixed to 150 fs. A_{λ,k_x} and τ_{λ,k_x} are the amplitude contribution and the time constant of each scattering processes. The fitted values for the variables were obtained by minimizing the squared sum of the difference between the measured data and the fitted function values via the MATLAB *fminsearch* algorithm, where the squared sum can be analytically expressed as:

$$\min\left[\sum_{\lambda,k_x}\left(\Delta OD_{\lambda,k_x}^{\mathrm{measured}}-\Delta OD_{\lambda,k_x}^{\mathrm{fit}}\right)^2\right] \qquad \text{(S11)}$$

9. Numerical filtering of coherent artifacts

An important aspect that requires consideration during the numerical fitting is minimizing the influence of the coherent artifacts present in the transient response. The coherent artifacts, which in our experimental data is mostly observed in the form of cross-phase modulation (XPM), show up as sharp and rapidly oscillating signals within the early time windows of the transient response which is not related to the hot-carrier-driven all-optical switching of the plasmonic grating. Therefore the presence of the coherent artifact hinders accurate analysis of the carrier-dynamic-induced time response near time zero, and the information of the temporal response, in the form of time constants, can be contaminated. This becomes increasingly important as we approach the nodal points where the recovery time gets faster and the coherent artifacts play a dominant role in the induced modulation depth. Since the amplitude of the transient signal from the coherent artifact is larger near the nodal points, the fminsearch algorithm will likely favor to fit the analytical function to the coherent artifact instead of the actual transient response of the sample. As a result, the contribution of the transient response from the sample remains ambiguous which can even lead to wrong interpretations.

In order to avoid such concerns, the influence of the coherent artifact was suppressed through a number of steps. First, a low pass filter and a moving average window was applied within the time window where the coherent artifact is existent to filter out the coherent artifact. Second, the instrument response function (which has a much longer time constant compared to the oscillation cycles of the coherent artifact) limits the time constants to capture the ultrafast oscillation coming from the coherent artifact. Last, generally, three time constants (effectively two since one is set to infinity) used for the fitting cannot fully capture the coherent artifact given that it shows multiple oscillations.

In order to demonstrate the effectiveness of the suppression scheme, we present a transient response curve of a glass substrate, where coherent artifacts are the only observable transient features as shown in Figure S8a. Repeating the numerical fitting, we see that the algorithm returns a fitted curve which is effectively a constant that equals to zero. Furthermore, after performing the numerical fitting throughout the wavelengths of interest, we plotted the recovery time constant as a function of wavelength, which shows no meaningful trend as observed in Figure S8b. The two results shown in Figure S8 well demonstrates the incapability of the fitting algorithm to capture the ultrafast oscillations, which signifies the effectiveness of the suppression scheme.

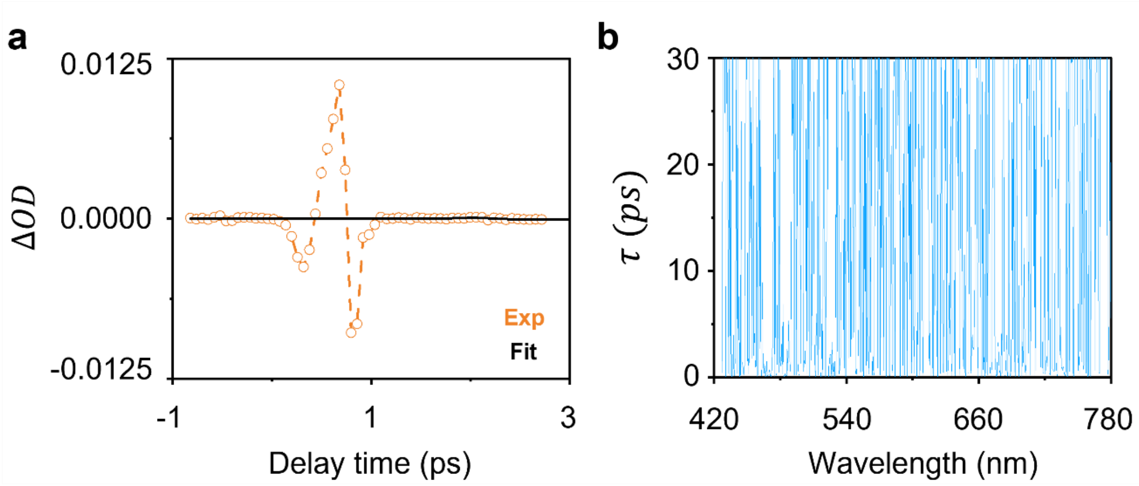

Figure S8 a) Experimental measurement of the coherent artifact at $\lambda = 589\ nm$ and the analytical function that best fits the measured data obtained through the fitting algorithm. b) Recovery time constants plotted as a function of wavelength. The experimental results by performing the pump-probe measurements on glass with the same excitation condition with a TE polarized probe light.

10. Origin of the ultrafast recovery time near the nodal points

In order to have a complete picture on the dispersive nature of the recovery time, a detailed discussion on the temporal dynamics of the plasmonic crystal near the nodal points is required. We first start by looking at the transient response curve at one of the probing conditions that gives a fast recovery time, which is given in Figure S9. Here, we see a rapid oscillation in a short time window which occurs due to the coherent artifact, and then we see a transient response which the modulation flips in sign as time passes. As we see in Figure S9, the short recovery time occurs when the early part of modulation has a larger amplitude compared to the amplitude after the transient response flips sign. Hence, effectively the recovery time becomes shorter than the time it takes for the transient response to flip in sign which is shorter than the relaxation timescale of hot-carriers. The given transient response, obtained after numerical fitting, gives us a recovery time constant of $\tau = 0.5528\, ps$, which corresponds to the exceptional points mentioned in the main text.

The origin of such nonlinear behavior lies within the nonlinear spectral profile of the resonance mode itself. Thus, a similar temporal behavior observed in the experiment can be analytically reconstructed with relative ease via a simple model, which excludes complex carrier-dynamic effects. Here, we employ a Lorentzian curve with time-varying spectral locations and linewidths. The values of the time-varying spectral locations and linewidths are assigned to follow an ex-gaussian profile with different time constants (3 ps and 2 ps respectively), where the time constant of the temporal change of the spectral location is larger than the time constant that describes the time evolution of the spectral linewidth. This is to mimic the effect where the relaxation of the modulation of the real part of the refractive index is slower than the imaginary part. Thereafter, we calculate the modulation in terms of ΔOD and plot the transient response

near the nodal point which is given at the right plot of Figure S9. A similar profile can be obtained even when we turn off the temporal evolution of either the spectral location or spectral linewidth, which suggests that the traits of carrier-dynamics - other than going through a rise and decay that qualitatively follows an ex-gaussian function - reflected in the model has less importance in reconstructing the transient response we see. Therefore, we deduce that the nonlinear characteristics of the spectral profile itself is the main cause of the ultrafast effect near the nodal points, which again confirms the role of optical resonances in the dispersion of recovery speed and emphasizes the importance of optical resonances in tailoring the temporal attributes of the hot-carrier-driven all-optical switch. The fact that we obtain a faster recovery time despite the assigned value for the characteristic timescale of the hot-carrier dynamics being much larger, further supports the capability of optical resonances accelerating the recovery speed of the transient response at specified conditions.

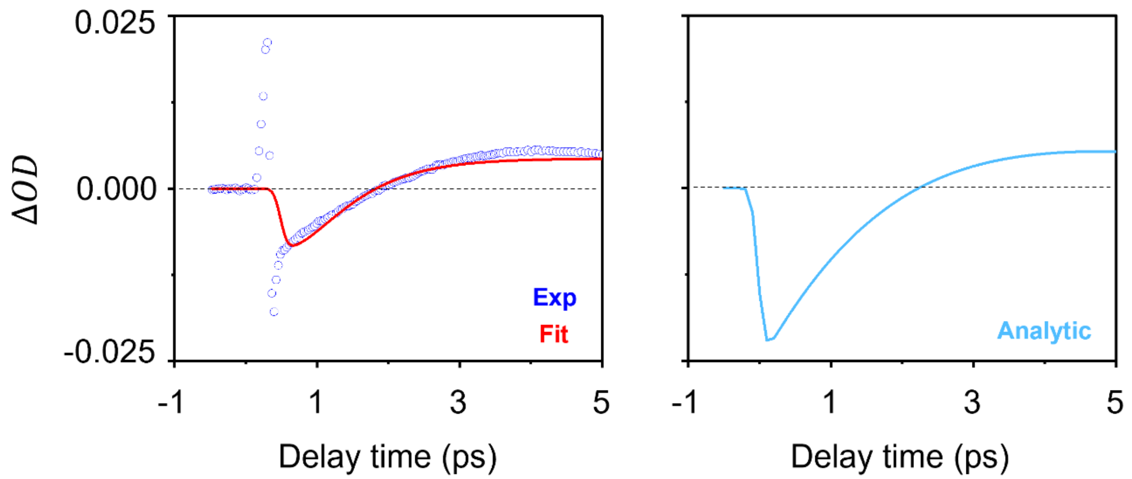

Figure S9 1 (Left) An example of the transient response observed near the nodal point (probe conditions were $\lambda = 589\ nm$, normal incidence, and TE polarization) and the analytical function fitted to the measurement. (Right) Analytical reconstruction of the temporal behavior near the nodal point with a simple model.

11. Photon lifetime as the origin for the dispersion of recovery speed

As part of an attempt to address the origin of the time constant dispersion (or longer recovery time near optical resonances) presented in Figure 4 in the main text, the characteristic photon lifetime of resonance modes has often been pointed responsible for such effect. Larger characteristic photon lifetime of a resonance mode leads to increased time required to form steady-state optical resonance in the resonator,[6] which thus causes a delay in the recovery of the optical response upon perturbation. For the case of hot-carrier excitation, the photon lifetime delays re-formation of the static resonance response following the complete recovery of hot-electron induced refractive index change. However, the impact maybe subtle as the spectrally broad nature of the resonance modes in our work leads to a relatively short photon lifetime, implying that this isn't the main factor of the slower modulation speed near resonance wavelengths. We take an $m = +1$ GMR mode at an AOI of 16° for example to estimate the timescale of the longest possible photon lifetime for the resonance modes supported by our sample. The photon lifetime can be obtained through the Q-factor of the resonance modes, which is calculated by $\lambda_0/\Delta\lambda$. As we see in Figure S9, λ_0 is the resonance wavelength which is located at 656 nm, and $\Delta\lambda$ is the full width at half maximum (FWHM) which is approximately 22nm. Therefore, we can estimate the Q-factor to be ~30 where and thus the photon lifetime is roughly ~10 fs which is much shorter than the timescale for the recovery speed. The photon lifetime τ_p was calculated by $Q = \omega_0\tau_p$ where ω_0 is the angular resonance frequency.

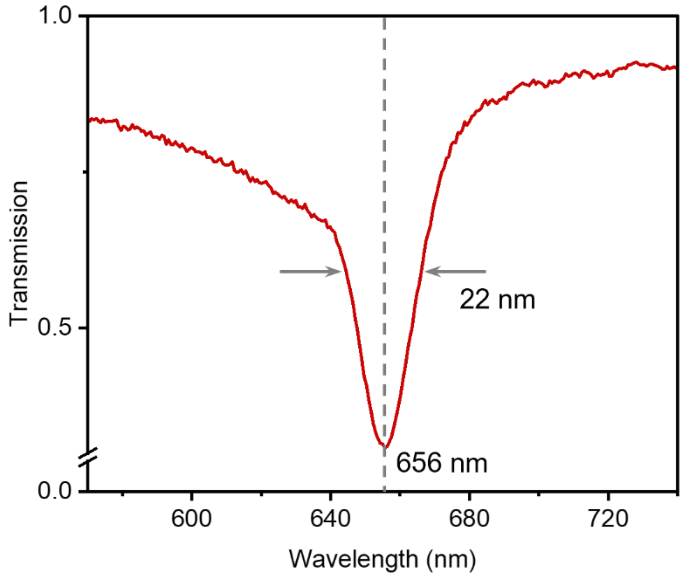

Figure S10. Optical transmission graph showing the resonance profile of a $m = +1$ GMR mode at an AOI of 16° under TE illumination. The resonance wavelength and FWHM is also shown together on the graph.

References

[1] M. Taghinejad, H. Taghinejad, S. T. Malak, H. Moradinejad, E. V. Woods, Z. Xu, Y. Liu, A. A. Eftekhar, T. Lian, V. V. Tsukruk, A. Adibi, *Ann. Phys. (Berl.)* **2018**, 530, 1700395.

[2] L. Rayleigh, *Proc. R. Soc. Lond. Ser. A* **1907**, 79, 399.

[3] H. P. Herzig, *Micro-optics: elements, systems and applications*, CRC Press, **1997**.

[4] A. Marini, M. Conforti, G. Della Valle, H. W. Lee, T. X. Tran, W. Chang, M. A. Schmidt, S. Longhi, P. S. J. Russell, F. Biancalana, *New Journal of Physics* **2013**, 15, 013033.

[5] A. Schirato, M. Maiuri, A. Toma, S. Fugattini, R. Proietti Zaccaria, P. Laporta, P. Nordlander, G. Cerullo, A. Alabastri, G. Della Valle, *Nat. Photonics* **2020**, 14, 723.

[6] M. Taghinejad, W. Cai, *ACS Photonics* **2019**, 6, 1082.
